# AFORS-HET-based numerical exploration of tunnel oxide passivated contact solar cells incorporating n- and p-type silicon substrates

**DOI:** 10.1039/d4ra03286a

**Published:** 2024-07-15

**Authors:** Rabia Saeed, Sofia Tahir, Adnan Ali, Hind Albalawi, Arslan Ashfaq

**Affiliations:** a Department of Physics, Government College University Faisalabad 38000 Faisalabad Punjab Pakistan sofetahir@gmail.com arslan.ashfaq201@gmail.com; b London Centre for Energy Engineering (LCEE), School of Engineering, London South Bank University London SE1 0AA UK; c Department of Physics, College of Sciences, Princess Nourah bint Abdulrahman University (PNU) P.O. Box 84428 Riyadh 11671 Saudi Arabia

## Abstract

The development of a tunnel oxide interfacial layer capped by a highly doped poly-Si layer is considered one of the most promising methods to reduce charge carrier recombination and improve the performance of conventional PERC devices. The thickness and doping concentration of emitters and BSF layers greatly influence the tunnelling current in TOPCon devices. In this research, we evaluated the performance of tunnel oxide passivated contact (TOPCon) solar cells by conducting an in-depth analysis of various key parameters. The parameter include the type of silicon substrate (n or p-type); the thickness and doping density (*N*_a_/*N*_d_) of n, n^+^, p, and p^+^ layers; and surface recombination velocity (front/rear), which were analyzed using AFORS-HET simulation software. A comparative analysis of performance demonstrates that the highest efficiency is achieved in the n-TOPCon solar cell with the following values: *V*_oc_ = 660.2 mV, *J*_sc_ = 45.05 mA cm^−2^, FF = 82.87%, and PCE = 25.74%. In the optimized p-TOPCon solar cell, the open circuit voltage (*V*_oc_) and fill factor (FF) exhibit improvements of 35.9 mV and 0.39%, respectively. However, the values of *J*_sc_ and PCE decrease by 6.44 mA cm^−2^ and 2.2%, respectively, in p-TOPCon solar cells. Furthermore, photo-electroluminescence analysis reveals that the n-TOPCon solar cells exhibit a higher maximum photon flux (front/rear) than p-TOPCon solar cells.

## Abstract 2

The development of a tunnel oxide interfacial layer capped by a highly doped poly-Si layer is considered one of the most promising methods to reduce charge carrier recombination and improve the performance of conventional PERC devices.

## Graphical abstract

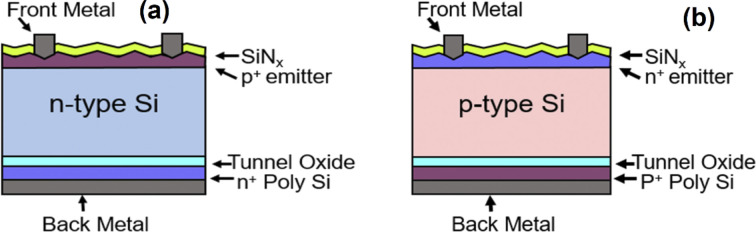

## Introduction

The silicon solar cells with a TOPCon structure are currently dominating the PV industry, according to report by ITRPV. However, the aluminum back surface field (AlBSF) technology is likely to cease after 2025. TOPCon silicon devices are considered innovative and are expected to eventually replace passivated emitter and rear cell (PERC) ideas in the future^1^ owing to their greater open circuit voltage (*V*_oc_) and higher fill factor. Presently, silicon devices with a tunnel oxide passivated contact cell (TOPCon) structure exhibit exceptional potential and have attracted considerable interest. This innovative idea avoids the somewhat complex process for localized contacts and provides the advantage of full-area contact.^2^ It is compatible with the current manufacturing line and high-temperature process conditions. In solar cells, a thin oxide layer is employed to offer complete-area passivation of greater quality, as well as an electron tunnelling function on the rear surface of the silicon wafer. There are also not many studies on the effects of silicon/di-electric layers to have a more comprehensive understanding of the performance of the devices, and to achieve successful full-area carrier collection, a highly doped n^+^/p^+^ polysilicon layer is often placed between a thin oxide layer and metal contact at the rear side.^3–5^

Despite a di-electric layer that passivates the rear side of a silicon passivated emitter and rear cell devices, recombination at the interface between the metal and silicon still occurs.^6,7^ In industrial PERC devices, which possess a performance of 22.21%, the current density of the recombination at the rear metal contact has been measured at 660 fA cm^−2^.^8,9^ Moreover, the contact geometry on the rear side of the PERC design, which is locally patterned, introduces 3D charge carrier transport. This leads to increased internal device resistance and decreases the fill factor of the cell.^10–12^ Additionally, the utilization of low-resistivity silicon wafers during PERC device production contributes to elevated levels of light-induced degradation (LID) and also light and elevated temperature-induced degradation (LeTID).^13–15^ LID is an ordinary process that leads to efficiency degradation of crystalline silicon solar cells. LID degrades rather quickly and can reach saturation in a few days. In recent years, the photovoltaic world has become aware of a new phenomenon causing crystalline silicon solar cells to degrade. It can be found in PERC solar cells made of monocrystalline and multi-crystalline silicon (mc-Si). This process is termed light and LeTID. The light-induced degradation process reaches saturation in a short period of time at the normal temperature, while LeTID requires high temperatures for a longer duration to reach saturation. LeTID will have substantial and long-term effects on the silicon photovoltaic modules.^16–18^ Currently, PERC solar cells lead the market, and LeTID can cause up to 16–17% performance loss in the PERC solar cells. Consequently, the study on light and LeTID is substantial. The results from the study of Bredemeier *et al.* demonstrate a relationship between the silicon wafer thickness and the degree of LeTID deterioration. The results obtained by Bredemeier *et al.* demonstrate that the degradation degree of LeTID is linked to the thickness of the silicon (Si) wafer. A Si substrate with less than 120 μm thickness does not suffer much degradation.^19,20^

In the TOPCon structure, charge carriers pass through insulators by a quantum tunneling process that passivates wafer and also reduces the recombination losses. The same apparatus that is used for the fabrication of PERC solar cells can also be used for manufacturing the TOPCon structure.^21,22^ The manufacturing of the TOPCon structure is more straightforward than that of the PERC solar cells, as there is complete passivation and metal contact at the rear side. During the fabrication route of TOPCon devices, thin layers of tunnel oxide and highly doped polycrystalline-silicon (poly-Si) are deposited, effectively minimizing the charge carrier recombination.^23,24^ The TOPCon structure has significant potential to penetrate the global market. The TOPCon solar cells offer several advantages over the traditional PERC solar cells, one of which is the presence of one-dimensional (1D) carrier flow facilitated *via* tunnelling through the oxide layer. In TOPCon structures, the silicon wafer is not directly in contact with the back metal but is parted by polysilicon and SiO_*x*_ layers. Highly efficient crystalline silicon solar cells have the tunnel oxide passivated contacts at the rear side to prevent recombination. The TOPCon solar cell, which stands for Tunnel Oxide Passivating Contact, operates under the assumption that carrier transport occurs *via* quantum tunneling through an ultrathin SiO_*x*_ layer (<2.0 nm). Despite rapid improvements in the TOPCon cell efficiency, some basic device physics remains poorly understood. SiO_2_ has long served as an insulating di-electric layer in thin-film transistors used in displays due to its well-known properties as a perfect insulator. However, photo-generated carriers in TOPCon solar cells must transport through an insulating SiO_*x*_ layer, and the transport mechanism is logically assumed to be tunnelling.

Another significant benefit is the superior carrier selectivity provided by the SiO_*x*_ layer. It achieves this by enabling drift currents of only one type of charge carrier *via* quantum mechanical tunnelling, thereby reducing minority charge carrier recombination at back contact.^23,25^ The thin oxide layer in TOPCon solar cells allows for efficient electron/hole transfer, depending on the conductivity of the substrates. This is due to dangling bonds, which are chemical bonds connected to atoms in the solid's surface layer that extend outward rather than joining with other atoms, on the top surface of the single crystal. The utilization of highly doped polysilicon, with its high conductivity, helps reduce junction resistance and enhance current output.^26^ These layer properties show crucial parts in cell design, aiming to achieve high *V*_oc_, greater efficiency and fill factor, all of which are promised by the TOPCon structure.

The effective growth of the TOPCon configuration hinges on four crucial steps: (1) the creation of an ultra-thin SiO_*x*_ layer, (2) the growth of a highly doped amorphous silicon onto SiO_*x*_ as the above-mentioned layer, (3) thermal annealing at high temperatures to activate dopants and crystallize the doped amorphous silicon layer, resulting in a multi-crystalline structure, and (4) subsequent hydrogenation handling after annealing to diminish defect states within the doped polycrystalline silicon layer.^27^ Therefore, it becomes evident that advancing and optimizing each of these treating steps can enable the production of high-performance TOPCon devices compared to the PERC configuration.

TOPCon devices are considered to be the next-generation technology for enhancing the performance of industrial silicon devices. The initial proposal for the structure of the TOPCon devices was made by Feldmann *et al.* in 2013, where a 10 × 10 cm^2^ cell achieved an efficiency of 22.9%.^28^ The recombination rate refers to the loss of generated charge carriers and mainly occurs in the surface and bulk areas of the device. The rear di-electric or passivated contact reduces recombination.^29^ Several parameters influence recombination, including effective minority charge carrier lifetime (*τ*_eff_), emitter recombination current (*J*_oe_), and surface recombination velocity (SRV). High SRV results in the formation of a dead layer when the SRV increases, with the carrier recombining at the defect surface owing to fewer minority carriers' lifetime and the diffusion length. The SRV is a crucial variable significantly affecting the efficiency of the devices. The SRV and the dead layer also impact the supplementary parameters of the cell, such as *J*_sc_ and *I*_sc_. The effective lifetime is determined by both the surface lifetime (*τ*_s_) and bulk lifetime (*τ*_b_), and their relationship can be explained using the following equation:1
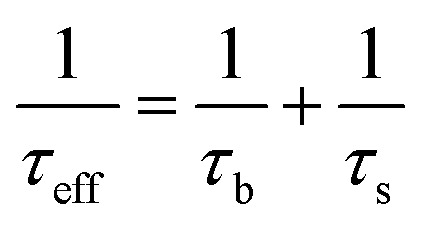


Effective charge carrier lifetime can be demonstrated as a function of SRV using the following equation:2
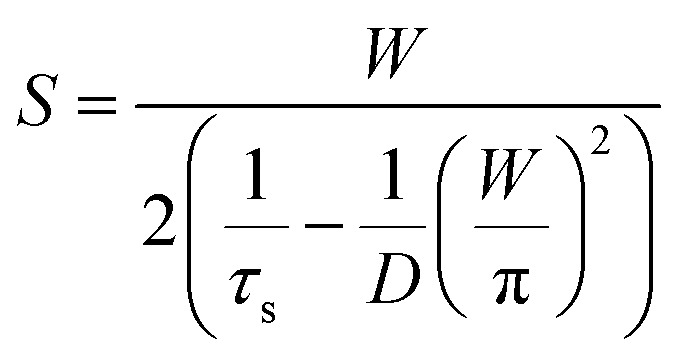
In these equations, *W* denotes the thickness of the silicon wafer and *D* denotes the diffusion constant of the minority carriers.

The efficiency of p-TOPCon cells was investigated by Quokka simulations, where variations in bulk lifetime (*τ*_b_), *ρ* (resistivity), and carrier discrimination of the polycrystalline silicon passivated contact were examined.^30^ Reducing surface charge recombination losses is fundamental to achieving enhanced efficiency in commercial solar cells. In the absence of illumination, the emitter recombination current surpasses the base recombination current. This is primarily attributed to the high defect density in the majority section, energy-band gap reduction, and the Auger recombination route.^31^

Fraunhofer ISE has recorded a record efficiency of 25.7% for these cells. Most studies on TOPCon structure solar cells have focused on utilizing SiO_2_ as the tunnelling layer on the n-type silicon wafers owing to its outstanding passivation properties for the n-type silicon interface. Moreover, marketable solar cells currently rely mainly on the p-type PERC structure. Transitioning from the p-PERC configuration to the p-TOPCon configuration necessitates a different di-electric layer for the tunnelling oxide layer. Nevertheless, the average stabilized performance of large-area n-TOPCon devices in mass fabrication has been found to exceed that of the prevailing p-PERC devices in the market.^32^

Several theoretical simulation works have explored using SiO_2_/polycrystalline silicon passivation contacts at the rear for p-TOPCon cells in the last few years. TCAD simulations have examined the impact of tunnelling oxide thickness and bulk properties on p-TOPCon cell performance.^33^ Gao *et al.* reported a simulation study of a 22.40% p-TOPCon structure using the Quokka software with industrial-grade process parameters.^34^ A logical, numerical study is mandatory for a deeper knowledge of significant factors that influence how well the devices perform. These fundamental characteristics are the quality and thickness of the wafer, BSF, emitter layer, the quality of the n^+^-Si layer and doping concentration. Steinkempe *et al.* has provided a useful example for numerical simulation to determine the performances of the TOPCon solar cells with several n^+^-Si materials.^35,36^

In this work, we studied the performance of TOPCon devices using both n- and p-type Si wafers. We varied the thickness of the emitter, substrate, and back surface field layers, as well as the doping density of the emitter/base/rear side (*N*_a_/*N*_d_) and the SRV. We aimed to optimize these parameters and achieve high efficiency for both n- & p-type TOPCon structures. We also assessed the impact of these parameters on the front and rear photon flux (measured in m^−2^ S^−1^) using the AFORS-HET simulation software.^37^

## Simulation methodology

**Fig. 1:**
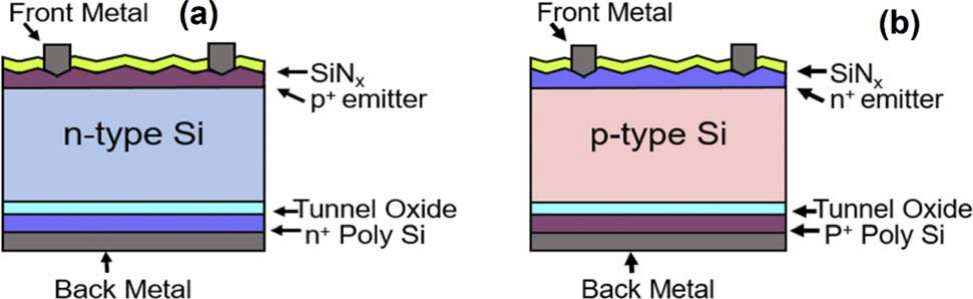
Simulated structure of (a) n-type and (b) p-type TOPCon devices.

Fig. 1 displays the schematic diagram of the n- and p-type TOPCon solar cell simulated using AFORS-HET. AFORS-HET employs finite differences to solve one-dimensional 1D semiconductor equations under various conditions, including Poisson's and electron and hole transport equations. These conditions include the following: (a) equilibrium mode to describe a semiconductor device numerically; (b) steady-state mode; (c) steady-state mode with minor sinusoidal perturbations; (d) basic transient mode, where external quantities are instantly turned on or off; and (e) general transient mode, which allows for changes in any external quantity. Furthermore, the software considers various physical processes of silicon devices, such as recombination, generation, transportation, and contact.^38^ AFORS-HET mathematically explains semiconductor eqn (3)–(5) and the suitable boundary limitation in one dimension.3
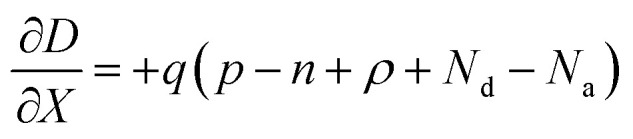
4
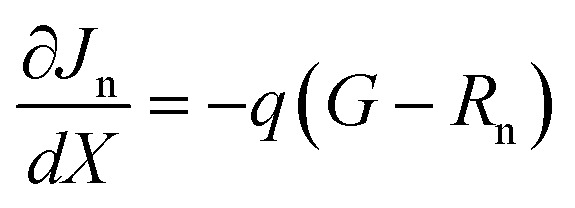
5
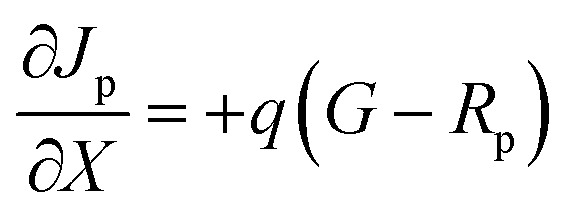
where *D* is the displacement field, *n* and *p* are the electron and hole densities, *ρ* is the net charge of all the traps positioned in the band gap, and *N*_a_ and *N*_d_ are the acceptor and donor densities. The electron and hole current densities are also indicated as *J*_n_ and *J*_p,_ respectively. *G* represents the optical generation rate, while *R*_n_ and *R*_p_ correspond to electron and hole recombination rates. Various recombination processes such as Auger recombination, direct band-to-band recombination, and the Shockley–Read–Hall recombination can be employed to model recombination phenomena.^39^ The electron and hole pair creation can be explained by considering the Lambert–Beer absorption or coherent and incoherent multiple reflections. Interface currents are modelled using either thermionic emission or drift-diffusion. Various models can represent metallic inter contacts, such as metal/insulator/semiconductor contacts or Schottky or Schottky Bardeen semiconductor/metal contacts.^40^ Many simulation articles have been published using AFORS-HET, which has gained widespread acceptance as a tool for understanding Si-heterojunction solar cells. The default parameters for optical and electrical layers optimized in this research are based on previously published works.^28^ The configuration of the n-TOPCon device used in this study is as follows: front electrode/SiN_*x*_/p^+^-Si/n-Si wafer/SiO_2_/n^+^-Si/back electrode. For the p-TOPCon device, the structure is as follows: front electrode/SiN_*x*_/n^+^-Si emitter/p-Si wafer/SiO_2_/p^+^-Si/back electrode.^41^ The input initial parameters used for the simulations can be found in Tables 1 and 2 for the *n* and p-TOPCon devices, respectively, and all optimization is carried out with the thickness of the tunnel oxide SiO_2_ layer at 1 nm for both n/p TOPCon solar cells.

**Table 1:** Input device parameters used for the simulation of the n-TOPCon solar cells

Designed structure layers	Optimized parameter
SiN_*x*_ di-electric	*t* = 70 nm
Boundary of the front contact	Texture surface (54.75°), fat band, absorption loss
Front metal contact	MS Schottky contact model, SRV = 10^2^ cm s^−1^
p^+^-type silicon layer	*t* = 0.1 μm, lifetime setting: 1 μs, *N*_a_ = 5 × 10^17^ cm^−3^
n-type single crystal silicon layer	*t* = 150 μm, *N*_d_ = 5 × 10^17^ cm^−3^, lifetime setting: 1 × 10^5^ μs
The interface of the SiO_*x*_	Chi = 1.0 eV, *d*_k_ = 3.9, *E*_g_ = 8.9 eV, *m*_h_ = 0.49, *m*_e_ = 0.98, *D*_ph_ = 0
n^+^-type silicon layer	*t* = 2 nm, setting of a lifetime: 50 μs, *N*_d_ = 7 × 10^20^ cm^−3^
Back contact	MS Schottky contact model, SRV = 1 × 10^4^ cm s^−1^
The boundary of back contact	Plane surface, flat band, w/o absorption loss
Ag electrode	*t* = 1 μm

**Table 2:** Input parameters used for the simulation of the *p*-TOPCon device

Layers	Optimized parameter
SiN_*x*_ di-electric	*t* = 75 nm
Boundary of front contact	Texture surface (54.75°), fat band, absorption loss, fat band
Front contact	MS Schottky contact model, SRV = 100 cm s^−1^
n^+^-type silicon layer	*t* = 0.1 μm, *N*_a_ = 2 × 10^18^ cm^−3^, lifetime setting: 1 μs
p-type c-Si layer	*t* = 100 μm, *N*_d_ = 2 × 10^18^ cm^−3^, setting of lifetime: 1 × 10^5^ μs
Interface of the SiO_*x*_	Chi = 1.0 eV, *d*_k_ = 3.9, *E*_g_ = 8.9 eV, *m*_h_ = 0.49, *D*_ph_ = 0, *m*_e_ = 0.98
p^+^-type silicon layer	*t* = 25 nm, *N*_d_ = 4.1 × 10^20 ^cm^−3^, setting of the lifetime: 50 μs
Back contact	MS Schottky contact model, SRV = 1 × 10^4^ cms^−1^
Boundary of the back contact	Plane surface, flat band, w/o absorption loss
Ag electrode	*t* = 1 μm

In the equations provided, *t* = thickness, *N*_a_ is the acceptor concentration, *N*_d_ is the donor concentration, Chi is the electron affinity energy, *E*_g_ is the energy band gap, *d*_k_ is the relative di-electric constant, *m*_e_ is the relative effective mass of the electron, *m*_h_ is the relative effective mass of the hole, and *D*_ph_ is the pinhole density through insulator layers.

## Results and discussion

**Fig. 2:**
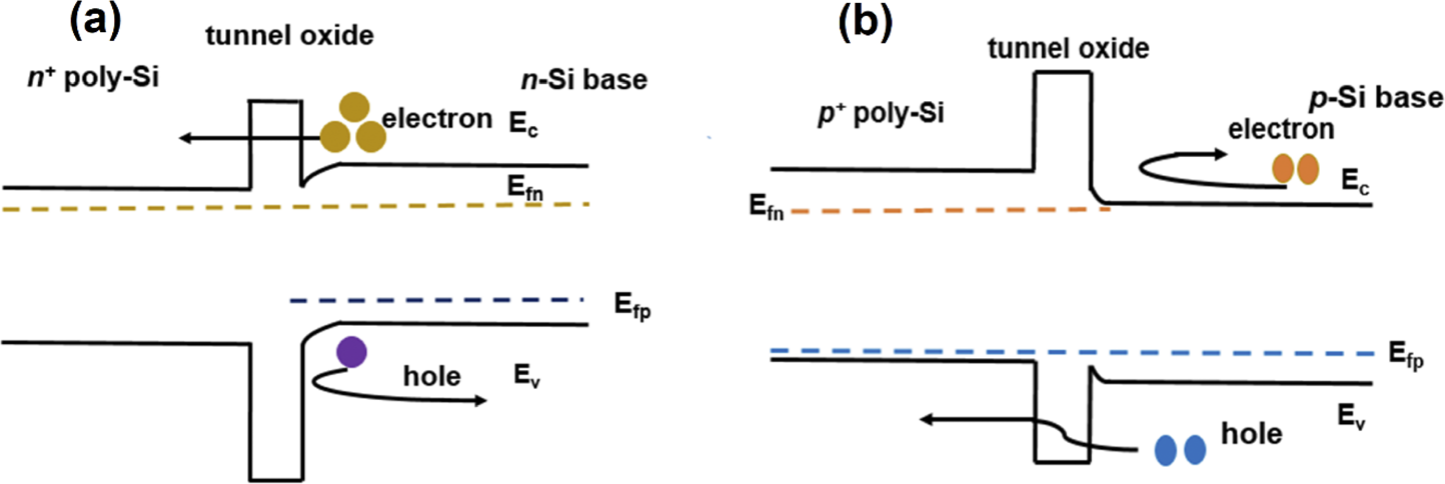
Energy band diagram showing the rear side of (a) n-TOPCon solar cells and (b) p-TOPCon solar cells.

To determine the device efficiency with different optimized parameters, The performance and photo-electroluminescence (m^−2^ S^−1^) of TOPCon structures were studied incorporating n- and p-Si substrates. The optimization of primary factors, namely, the thickness and doping density of the layers (n, n^+^, p, p^+^) and SRV (front/rear) was investigated here to increase the performance of n/p-TOPCon solar cells. The band diagram of both the p/n tunnel oxide passivated contact structures is shown in Fig. 2. In Fig. 2a, the band diagram of the rear side of n-type TOPCon is shown with the n-Si/SiO_2_/n^+^-poly-Si structure. First, heavily doped n^+^ poly-Si creates an accumulation layer at the absorber surface due to the work function difference between the n^+^ poly-Si and the n-Si absorber. This accumulation layer or band bending provides a barrier for holes to get to the tunnel oxide, while electrons can migrate easily to the oxide/Si interface that is favorable for the lesser barrier height of oxides at the conduction band as well as lesser electron effective mass at the SiO_2_ layer. Fig. 2b shows the p-Si/SiO_2_/p^+^-poly-Si structure in p-type TOPCon solar cells, featuring a lower valence band offset (Δ*E*_v_) and a higher conduction band offset (Δ*E*_c_) between p-Si and p^+^-poly-Si. This configuration facilitates hole collection on the heavily doped p^+^-poly-Si side.

### Path towards cell parameter optimization (n/p TOPCon solar cells)

The optimization of layers (thickness, doping concentration, and FSRV/RSRV) in the n-TOPCon solar cell was started from the silicon substrate layer. Then BSF and emitter layers were optimized gradually. The layer thickness/doping concentration optimization sequence is as follows: n-Si substrate, n^+^ Si layer, p^+^ Si layer, FSRV and then RSRV of the *n*-TOPCon structure, as given in Table 3. However, for p-TOPCon, it is p-type c-Si, p^+^-type Si layer, n^+^-type Si layer, FSRV and RSRV, respectively, as given in Table 4.

**Table 3:** Simulated n-TOPCon solar cells

Layers	Optimized value	Output parameters				
		*V*_oc_ [mV]	*J*_sc_ [mA cm^−2^]	FF [%]	*η* [%]	Con. *η* [%]
*n*-type c-Si	*t* = 150 um	527.3	37.88	70.83	14.51	1.1
	*N*_d_ = 5 × 10^17^ cm^−3^	655.5	34.49	74.35	15.34	0.755
n^+^-type Si layer	*t* = 2 nm	655.5	31.56	74.36	15.38	0.757
	*N*_d_ = 10 × 10^20^ cm^−3^	653.9	42.69	81.04	22.62	1.01
p^+^-type Si layer	*t* = 0.1 μm	657	44.48	81.25	24.81	1.02
	*N*_a_ = 5 × 10^17^ cm^−3^	660.2	45.05	82.87	25.74	1.03
FSRV	100 cm s^−1^	660.2	45.01	82.87	25.74	1.03
RSRV	10 000 cm s^−1^	660.2	45.02	82.87	25.74	1.03

**Table 4:** Simulated p-TOPCon solar cells

Layers	Optimized value	Output parameters				
		*V*_oc_ [mV]	*J*_sc_ [mA cm^−2^]	FF [%]	*η* [%]	Con. *η* [%]
p type c-Si	*t* = 100 μm	536.7	33.77	72.57	13.15	0.86
	*N*_d_ = 2 × 10^18^ cm^−3^	688.3	32.9	82.06	18.58	0.769
p^+^-type Si layer	*t* = 25 nm	688.3	32.9	82.06	18.58	0.772
	*N*_d_ = 4.1 × 10^20^ cm^−3^	688.3	35.42	82.07	20	0.83
n^+^-type Si layer	*t* = 0.1 μm	691.4	40.34	82.21	22.93	0.867
	*N*_a_ = 2.1 × 10^18^ cm^−3^	694.4	40.51	80.79	23.51	0.87
FSRV	100 cm s^−1^	696.1	40.61	83.26	23.54	0.87
RSRV	10 000 cm s^−1^	696.1	40.61	83.26	23.54	0.87

### Optimization of n-TOPCon solar cells

**Fig. 3:**
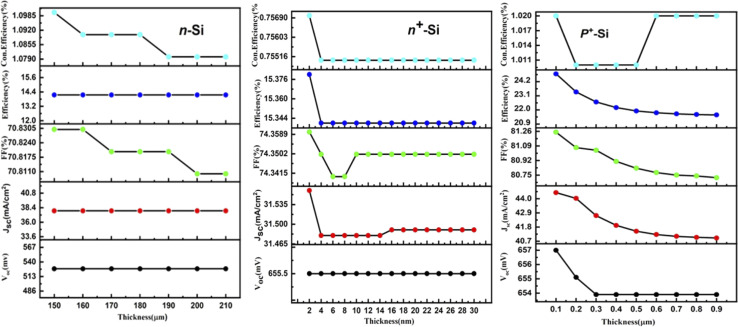
Effect of the thickness of n-Si, n^+^-Si and p^+^-Si on the *V*_oc_, *J*_sc_, FF, efficiency and con. efficiency of the n-TOPCon device.

**Fig. 4:**
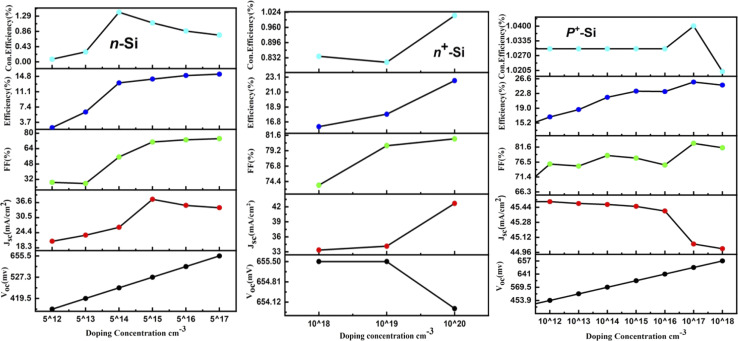
Effect of the doping concentration of n-Si, n^+^-Si and p^+^-Si on the *V*_oc_, *J*_sc_, FF, efficiency and con. efficiency of the n-TOPCon device.

At first, the silicon wafer thickness of the n-TOPCon solar cell was optimized by varying it from 150 to 210 μm, as presented in Fig. 3. A maximum efficiency of 14.15% observed at 150 μm reduction in wafer thickness will reduce the recombination sites, hence 150 μm was chosen as the standard wafer thickness. It was obvious that the FF and Con. efficiency decreased with the increase in n-Si wafer thickness. The results revealed that *J*_sc_, *V*_oc_, FF and *η* negatively correlated with the substrate thickness. Then the doping concentration of 150 μm-thick n-Si wafer was changed from 5 × 10^12^ to 5 × 10^17^ cm^−3^. A 0.83% increase in the efficiency of n-TOPCon was observed at a doping concentration of 5 × 10^17^ cm^−3^ for the n-Si substrate. The bulk doping level significantly affects bulk recombination in wafer-based TOPCon cells, as high doping levels control the minority carrier lifetime. Therefore, optimizing both the bulk doping level and wafer thickness is crucial for enhancing overall cell performance. An increase in *V*_oc_ (mV) and FF (%) and a decrease in *J*_sc_ (mA cm^−2^) were also observed in this case, as shown in Fig. 4. By using the optimized values of the n-Si wafer (thickness and doping concentration), the thickness of the BSF layer (n^+^ Si) was varied from 2 to 30 nm. It was observed that the efficiency increased by 0.04% with the increase in other output parameters at 2 nm thickness of the n^+^ Si BSF layer, as shown in Fig. 3. The optimized doping concentration of BSF (n^+^ silicon) was observed as 10 × 10^20^ cm^−3^ with the above-stated optimized parameters, as shown in Fig. 4. The result showed a certain increase in efficiency by +7.24%, as higher doping concentrations in the polysilicon layer reduce the series resistance of the cell. This improved conductivity allows for more efficient carrier transport reducing resistive losses and thus enhancing the fill factor and overall efficiency of the cell along with the increase in *J*_sc_ and FF of n-TOPCon. An increase in *J*_sc_ and efficiency with the increase in BSF doping concentration is due to a low recombination current. The increase in *V*_oc_ is also due to the additional built-in voltage that is produced by the BSF layer, so the *V*_oc_ value increases with the increase in doping value and the generation of electric field on the back surface of the cell, and minority charge carriers generated near the surface escape from the recombination process by this electric field.^42^

**Fig. 5:**
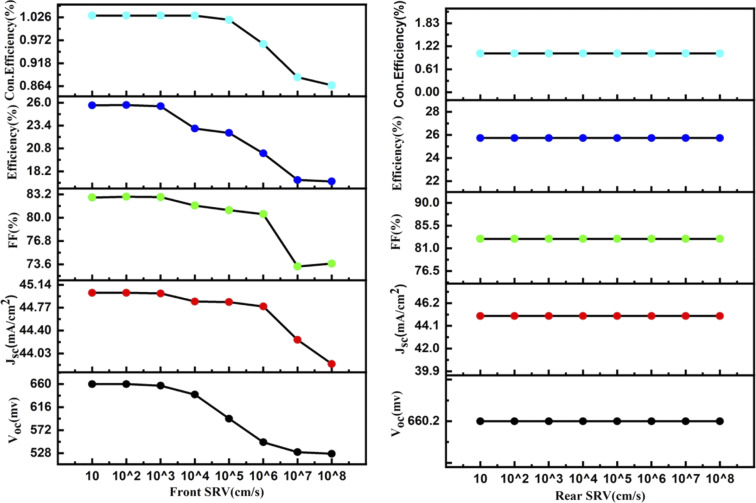
Effect of the front and the rear SRV on the efficiency of the n-TOPCon device.

By using the above-optimized parameters and the thickness variation of the p^+^ Si emitter layer from 0.1 to 0.9 μm, an increase in efficiency by +2.19% was observed for 0.1 μm p^+^ emitter thickness, as illustrated in Fig. 3. The doping concentration (p^+^ Si emitter layer) of 5 × 10^17^ showed a rise of +0.93% in efficiency along with the increase in *V*_oc_ and FF of simulated n-TOPCon devices, as shown in Fig. 4. The open circuit voltage of the cell, *V*_oc_, increased with the increase in the doping density in p^+^ Si, but short circuit current density, *J*_sc_, started to decrease. With the increase in doping density in p^+^ Si, the built-in potential increases, and the open-circuit voltage is related to the built-in potential and the quasi-Fermi levels. As the doping concentration increases, the built-in potential increases, which directly enhances *V*_oc_; meanwhile, the short circuit current density *J*_sc_ decreases. However, substantial excessive doping can also increase the recombination loss in the emitter region, increasing the recombination loss of light-induced carriers and reducing the number of free carriers available to contribute to the short-circuit current with decreased *J*_sc_.^43^ Therefore, the efficiency of the cell exhibited a maximum value at 1 × 10^17^ cm^−3^.^44^ The effects of front and rear SRV were also investigated in this work, as shown in Fig. 5. At first, we used a fixed rear SRV of 1 × 10^5^ cm s^−1^ and varied the front SRV from 1 × 10^1^ to 1 × 10^8^ cm s^−1^. The results showed the stable and maximum output parameters of *n*-TOPCon solar cells for 100 cm s^−1^ front SRV. However, the increase in front SRV decreased the *J*_sc_, FF and efficiency. Then by using 100 cm s^−1^ as the front SRV, the rear SRV was varied from 1 × 10^1^ to 1 × 10^8^ cm s^−1^. The result showed that the maximum *J*_sc_, FF and efficiency of *n*-TOPCon were observed at 1 × 10^4^ cm s^−1^ rear the SRV with 1 nm oxide thickness. However, the output parameters of the n-TOPCon device decreased with the increase in the front SRV but not much affected by the rear SRV, and it has been proved that an oxide of 1.0 nm thickness is sufficient to keep the *V*_oc_ and *J*_sc_ stable even when the rear SRV reaches 1 × 10^7^ cm s^−1^ and this might be because the oxide suppresses the leakage current,^3,45^ as depicted in Fig. 5. Simulation outcomes demonstrate that the conversion efficiency of silicon-based n TOPCon solar cells decreases as the SRV value increases owing to a larger recombination rate of the minority carriers near the front surface of the solar cell. The SRV leads to carriers that produce a loss added to the potential current or voltage of the cell as most of the recombination occurs in the majority and surface region, and a dead layer is produced due to the high SRV.^3^ Higher surface recombination velocity causes a decrease in the rate of photo-generated charge carriers. The detailed optimization effect of the n-TOPCon device is given in Table 3.

### Optimization of the p-TOPCon solar cell

**Fig. 6:**
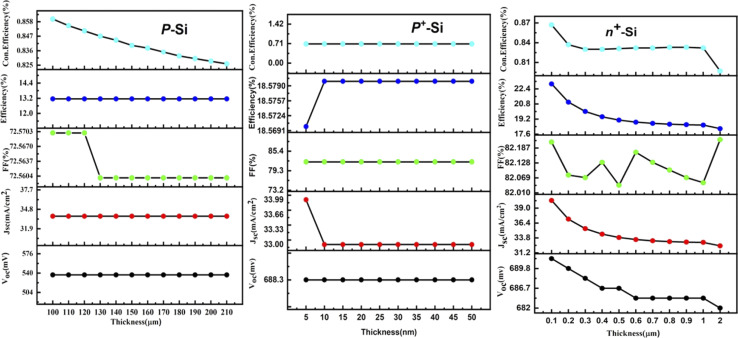
Effect of the thickness of p-Si, p^+^-Si and n^+^-Si on the *V*_oc_, *J*_sc_, FF, efficiency and Con. efficiency of the p-TOPCon device.

**Fig. 7:**
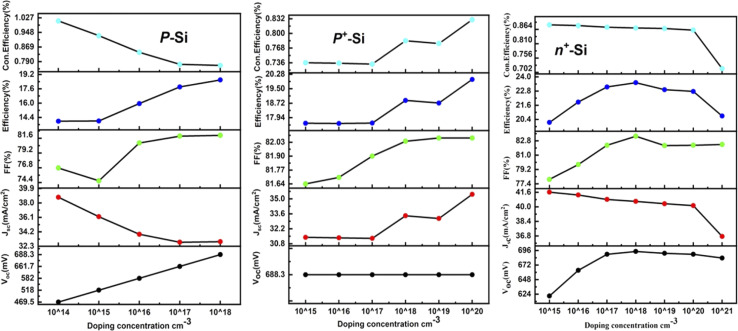
Impact of the doping density of p-Si, p^+^-Si and n^+^-Si on the efficiency of the p-TOPCon device.

Improvement of TOPCon configuration using p-type silicon wafers, similar to the p-PERC solar cell, is an appealing approach in photovoltaics. In the p-TOPCon structure, the emitter is made of n-type silicon that exhibits better surface properties than p-type silicon. Therefore, it is positioned at the front of the cell where most light is absorbed. Consequently, adopting the approach of having the top of the cell as the negative terminal and the rear as the positive terminal proves to be advantageous in p-TOPCon devices. In this work, we optimized the p-Si wafer thickness by varying it from 100 to 210 μm and selected it as 100 μm. The performance of p-TOPCon was 13.15% and *J*_sc_ was 33.77 mA cm^−2^, as depicted in Fig. 6. The Con. Efficiency and FF were found as maximum at this thickness, whereas all other output parameters were almost constant. The final photocurrent and overall cell performance depend highly on the silicon wafer's thickness. Although the cost is reduced when the thickness decreases, the production line's yield also decreases. One benefit of going thinner is that the lifetime does not become an issue; the thinner the layer, the lesser the possibility for carrier recombination. Then, using a 100 μm p-Si wafer, its doping concentration varied from 1 × 10^14^ to 9 × 10^18^. The doping density essentially affects the performance of solar cells. The reverse saturation current reduces with the increase in the doping density, enhancing *V*_oc_ and PCE. This improvement is continued until high doping effects begin to appear. Maximum doping levels affect the lessening of bandgap and minority charge carrier lifetime. Both cause the reverse *I*_sc_ to decrease again after reaching a peak with a doping density in the substrate. An increase of +5.43% in p-TOPCon efficiency was observed at 2 × 10^18^ cm^−3^ doping concentration (p-Si wafer) along with the increase in *V*_oc_ and FF, as shown in Fig. 7. We used the above-optimized parameters and varied the thickness of the BSF (p^+^ type silicon) layer from 5 to 50 nm, as shown in Fig. 6. Maximum efficiency with no increase in other parameters as compared to the earlier result was obtained at 25 nm. By producing a p^+^ layer under metallization, a back surface field is achieved. This layer reflects the minority carrier electrons and stops them from reaching the highly recombining metal–silicon contact, decreasing the minority carrier recombination, which essentially improves efficiency. By using 25 nm as the optimized thickness of p^+^ Si, its doping concentration was varied from 2 × 10^15^ to 9 × 10^20^ cm^−3^, as shown in Fig. 7. An increase of +1.42% in efficiency was observed at 4.1 × 10^20^ cm^−3^ with the increase in *J*_sc_ and FF. The back surface field (BSF) can be created by introducing a highly doped p^+^-Si layer near the rear surface of p-type bulk. As the acceptor doping level or the hole density increases, the energy of conduction and valence electrons also increases. This generates an electrical field (BSF) that pushes electrons towards bulk and attracts holes into the BSF area. This leads to better separation of electrons and holes close to the rear surface, reducing the electron–hole recombination and improving the overall efficiency.^46^

**Fig. 8:**
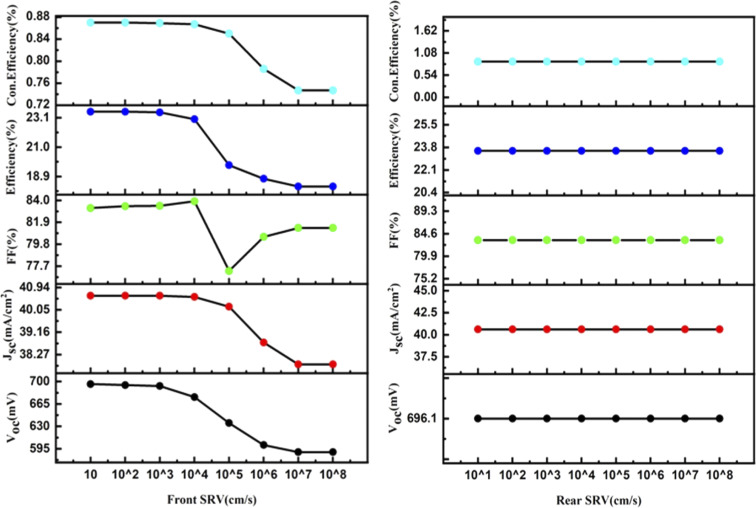
Effect of the front and rear SRV on the efficiency of the p-TOPCon device.

By using the above-optimized parameters, the thickness of the n^+^ Si emitter layer was varied from 0.1 to 2 μm, as shown in Fig. 6. A prominent increase of 2.93% in the efficiency of p-TOPCon solar cells was observed at 0.1 μm thickness of the n^+^ Si emitter layer because by making the front layer exceedingly thin, a significant portion of the carriers produced by the incoming light are absorbed near the front surface. Then the doping density of the n^+^ emitter layer was varied from 1 × 10^15^ to 9 × 10^21^ cm^−3^ by using the above-optimized parameters, as depicted in Fig. 7. An ideal emitter configuration aims to minimize the (i) recombination losses in the diffused region also at the surface of the cell and (ii) resistive losses as the emitter doping profile significantly affects the device characteristics of the solar cell.^47^ The performance of the p-TOPCon device was increased by 0.58% at 2 × 10^18^ cm^−3^ doping concentration (n^+^-Si) of the emitter layer that reduces reverse saturation current, thereby increasing the open circuit voltage. The *I*–*V* results show that *V*_oc_ and *J*_sc_ are intensely affected by FSRV and show no effects by changing the BSRV on P-TOPCon solar cells, as shown in Fig. 8, because carrier-selective connections are used in TOPCon cells to permit the electrons to flow through while blocking the holes. This selective transport mechanism reduces the recombination of minority carriers at the rear surface, thereby minimizing the effect of rear SRV on cell performance.^48^

By changing the FSRV both *V*_oc_ and *J*_sc_ decrease with the increase in SRV. A reduction in *J*_sc_ and *V*_oc_ is noticed only due to intense carrier recombination close to the front surface of the cells, which significantly lowers cell performance. Optimized front and rear SRVs are found as 100 cm s^−1^ and 10 000 cm s^−1^, respectively, with an improved efficiency of 23.54% of p-TOPCon devices.

The comparison of n-TOPCon and p-TOPCon showed a substantial increase in *V*_oc_ and FF of p-TOPCon devices. However, the efficiency and *J*_sc_ of n-TOPCon were higher than those of the p-TOPCon device.

Tables 3 and 4 show that the performance of the n/p TOPCon structure has gradually improved with the optimization of the thickness and doping density of all layers.

### Photo electroluminescence of n/p TOPCon

**Fig. 9:**
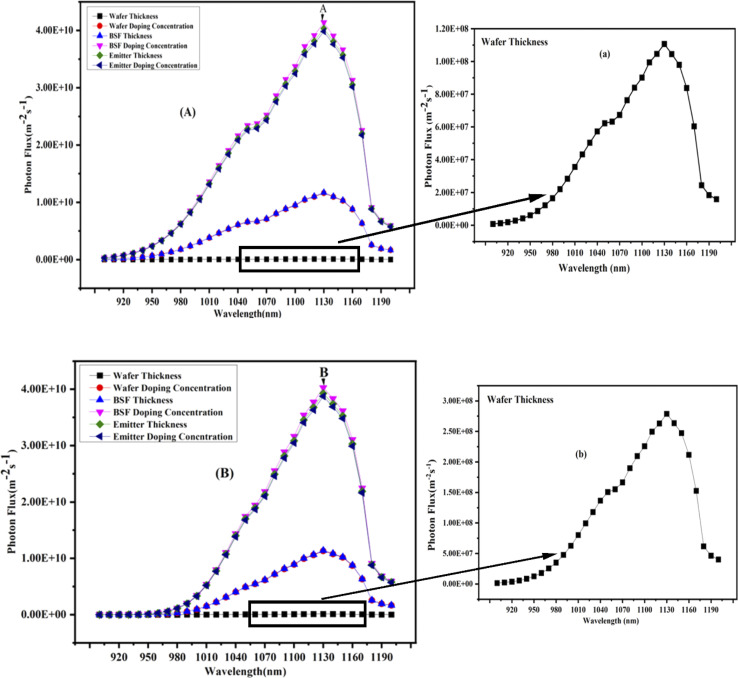
(A and B) Photon flux at the front and back sides of the n-TOPCon device.

**Fig. 10:**
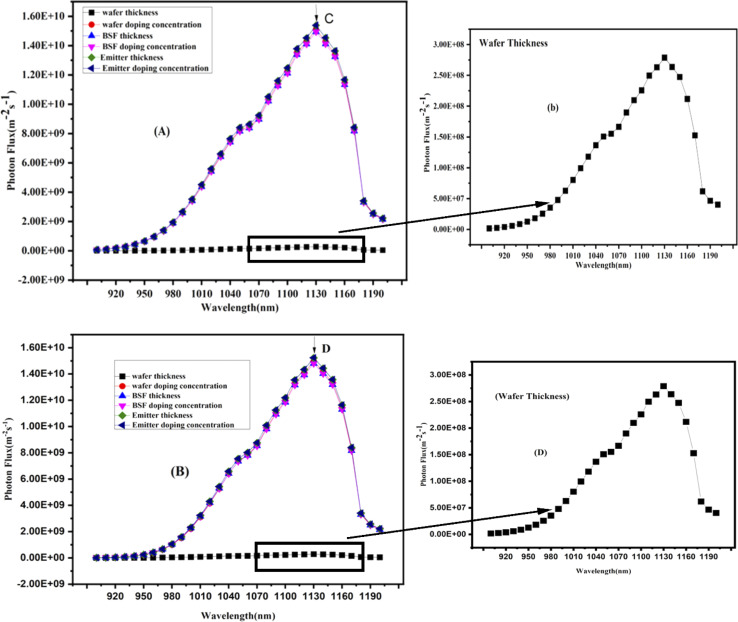
(C and D) Photon flux at the front and back sides of p-TOPCon devices.

Photoluminescence (PL) refers to light emission from a material when it is optically excited. When a material is exposed to light with sufficient energy, it absorbs photons and undergoes electronic excitations. Ultimately, these excitations relax and electrons return to their ground state. If this relaxation process involves light's radiative emission, it is known as photoluminescence. The nature of optical excitation plays a vital role in PL. The energy of excitation determines the initial photo-excited state and influences the depth to which the incident light can penetrate the material.^49^ The photon flux plays a crucial role in determining the number of generated electrons and, consequently, the current produced by solar cells. However, the photon flux alone does not provide information about the energy or wavelength of photons. Therefore, it is necessary to specify the energy or wavelength of photons in the light source. Combining the photon wavelength or energy with the corresponding photon flux can calculate the power density for the photons at particular wavelengths. Fig. 9 and 10 show the photoluminescence (PL) results for the n/p TOPCon solar cells. The figure and wafer thickness show a straight line in the PL spectrum. However, the actual intensity of the wafer spectrum is low as compared to the other parameters of the cell, as shown in the spectrum of the adjacent figure for both n/p TOPCon.

### Photo electroluminescence of n-TOPCon

The PEL results show a relation between the wavelength of the emitted light and the photon flux (m^−2^ S^−1^). Emission at longer wavelengths represents the transition of electrons with lower energy levels and *vice versa*. The photon flux is important in calculating the number of generated electrons and the current from n-TOPCon. Fig. 9 shows the normalized PL spectra (front/rear) of n-TOPCon over the wavelength range of 900–1200 nm. It was observed that the photon emission started near 900 nm wavelength, and then the photon flux tended to increase toward longer wavelengths. The peak values denoted by A and B showed maximum values of 4.1388 × 10^1^ m^−2^ s^−1^ and 4.028 × 10^1^ m^−2^ s^−1^ for the front and rear-side photon fluxes, respectively, at 1130 nm wavelength of light. There is a minor difference in the peak of n/p TOPCon, but the front surface photon flux is large due to more photon absorption than the rear side. Moreover, the short-circuit current density depends on the photon absorption of the front layer, and the Fill Factor (FF) is calculated for the balanced charge transport and the recombination properties of the material. The maximum photon flux of the n-TOPCon solar cell was obtained at the infrared wavelength range. The photon flux's peak value strongly depended on the optimized doping concentration (10 × 10^20^ cm^−3^) of the BSF (n^+^ Si) in the n-TOPCon solar cell. The BSF layer, either n^+^ or p^+^, enhances photon flux at the front/rear of the device, thus increasing the performance. The figure shows the wavelength dependence of photon flux, percentage of total photon flux, and the corresponding wavelength.

### Photo electroluminescence of p-TOPCon

Fig. 10 illustrates the normalized PL spectra (front/rear) of the p-TOPCon solar cell over a wavelength range of 900–1200 nm. Photon emission started near 900 nm wavelength, and then the photon flux increased toward longer wavelengths. The peak values denoted by C and D showed the maximum value of flux 1.538 × 10^1^ m^−2^ s^−1^ and 1.524 × 10^1^ m^−2^ s^−1^ for the front and rear-side photon flux, respectively, at 1130 nm wavelength of light that is maximum for the front surface of the cell; at points C and D for front/back photon flux, the maximum photon flux occurs for the doping concentration of the emitter layer, and emitter layer absorbs more photon. It renders better efficiency to the cell and increases the cell's short-circuit current. It is obvious that the maximum photon flux of the p-TOPCon solar cell was obtained at the infrared wavelength range The peak value of the photon flux was strongly dependent on the optimized doping concentration (2.1 × 10^18^ cm^−3^) of the emitter (n^+^ Si) in the p-TOPCon solar cell. This emitter layer (n^+^ Si) enhances the photon flux at the front/back of the device, thus increasing the efficiency. The results show that the photon flux (m^−2^ s^−1^) for the front and rear sides in n-TOPCon cells is higher than that in p-TOPCon devices. The number of electron–hole pairs formed is proportional to the number of photons absorbed by the cell. The optimization of new parameters in the TOPCon solar cell was increased the overall device performance.

## Conclusion

This study used the AFORS-HET numerical simulator to simulate TOPCon devices with n- and *p*-type silicon substrates. We aimed to optimize the device parameters and achieve a PCE of more than 25% in *n*-TOPCon cells and more than 23% in *p*-TOPCon cells. We optimized various parameters including layer thickness (Si-substrate, emitter, BSF), acceptor and donor doping concentrations (*N*_a_/*N*_d_), and the front and rear SRV. We optimized a doping concentration of 5 × 10^17^ cm^−3^ for the p^+^-type Si layer (0.1 μm), and obtained the following results for the *n*-TOPCon cell: *V*_oc_ = 660.2 mV, *J*_sc_ = 45.05 mA cm^−2^, FF = 82.87%, and PCE = 25.74%. A comparison between n-TOPCon and p-TOPCon cells revealed a significant increase in the *V*_oc_ and FF for the p-TOPCon cell. However, the efficiency and *J*_sc_ of the n-TOPCon cell were higher than those of the p-TOPCon cell. Furthermore, the maximum photon flux was observed in the *n*-TOPCon cell compared to the p-TOPCon cell. Photo electroluminescence (PEL) has direct effects on the power generation performance of solar photovoltaic cells. It is concluded that the open circuit voltage and short-circuit current of the TOPCon cell gradually increase with the increase in light intensity. Maximum output power increases with the maximum photon that falls on the cell and also increases the number of electrons produced; hence, more current is produced from solar cells. This theoretical research provides a foundation for developing efficient *p*-TOPCon solar cells with maximum performance, opening up new avenues for further advancements in this field.

## Author contributions

Rabia Saeed: software, writing, original draft; Sofia Tahir: writing, review & editing, supervisor; Hind Albalawi: formal analysis; Adnan Ali: visualization, conceptualization; Arslan Ashfaq: writing, original draft, writing, review & editing, conceptualization.

## Conflicts of interest

There are no conflicts to declare.
